# Highly Stretchable, Elastic, and Sensitive MXene-Based Hydrogel for Flexible Strain and Pressure Sensors

**DOI:** 10.34133/2020/2038560

**Published:** 2020-07-14

**Authors:** Yao Lu, Xinyu Qu, Wen Zhao, Yanfang Ren, Weili Si, Wenjun Wang, Qian Wang, Wei Huang, Xiaochen Dong

**Affiliations:** ^1^Key Laboratory of Flexible Electronics (KLOFE) & Institute of Advanced Materials (IAM), School of Physical and Mathematical Sciences, Nanjing Tech University (NanjingTech), 30 South Puzhu Road, Nanjing 211800, China; ^2^School of Physical Science and Information Technology, Liaocheng University, Liaocheng 252059, China; ^3^Shaanxi Institute of Flexible Electronics (SIFE), Northwestern Polytechnical University (NPU), Xi'an 710072, China; ^4^School of Chemistry and Materials Science, Nanjing University of Information Science & Technology, Nanjing 210044, China

## Abstract

Electronic skin is driving the next generation of cutting-edge wearable electronic products due to its good wearability and high accuracy of information acquisition. However, it remains a challenge to fulfill the requirements on detecting full-range human activities with existing flexible strain sensors. Herein, highly stretchable, sensitive, and multifunctional flexible strain sensors based on MXene- (Ti_3_C_2_T_*x*_-) composited poly(vinyl alcohol)/polyvinyl pyrrolidone double-network hydrogels were prepared. The uniformly distributed hydrophilic MXene nanosheets formed a three-dimensional conductive network throughout the hydrogel, endowing the flexible sensor with high sensitivity. The strong interaction between the double-network hydrogel matrix and MXene greatly improved the mechanical properties of the hydrogels. The resulting nanocomposited hydrogels featured great tensile performance (2400%), toughness, and resilience. Particularly, the as-prepared flexible pressure sensor revealed ultrahigh sensitivity (10.75 kPa^−1^) with a wide response range (0-61.5 kPa), fast response (33.5 ms), and low limit of detection (0.87 Pa). Moreover, the hydrogel-based flexible sensors, with high sensitivity and durability, could be employed to monitor full-range human motions and assembled into some aligned devices for subtle pressure detection, providing enormous potential in facial expression and phonation recognition, handwriting verification, healthy diagnosis, and wearable electronics.

## 1. Introduction

In recent years, electronic skins (E-skins) have attracted extensive interests due to their similar functions to the human skin, including stretchability, multifunctional sensing capabilities, and wide sensing range [1]. Among them, flexible strain sensors convert physiological activity signals into visible electrical signals in the form of signal transmission, exhibiting great potential in flexible touch screens, health clinical monitoring, industrial robots, and so on [2]. Elastic polymer substrates are widely utilized for the fabrication of stretchable electronics, such as polydimethylsiloxane, polyurethane, and poly(ethylene terephthalate), which manifest high transparency, a certain degree of elasticity, and good stability [3–5]. However, the limited tensile properties and low sensitivity severely impede their practical applications in flexible strain sensors.

Hydrogel, a three-dimensional (3D) networked structure containing a large amount of water or ionic liquid, has been widely explored for flexible strain sensors for its excellent stretchability, surface compliance, and biocompatibility [6, 7]. However, with limited degree of crosslinking and high viscosity, single-network hydrogels exhibit poor mechanical property and stability [8, 9]. This problem can be effectively alleviated by introducing effective energy dissipation domains or constructing a double-network hydrogel [10–12]. Nevertheless, it is still challenging to combine good mechanical property with high sensing performance to design a highly stretchable and sensitive strain sensing hydrogel for wearable electronics. Recently, conductive materials, such as conductive polymers, carbon nanomaterials, and metal nanoparticles, have been incorporated into a polymer matrix to improve the electromechanical performances of hydrogel-based strain sensors [13–15]. For example, carbon nanomaterials could greatly increase the elongation of the hydrogel due to strong interaction between their rich surface groups and polymer skeleton. Meanwhile, they also could dramatically improve the electrical conductivity of the hydrogel, thus significantly enhancing the sensitivity of strain sensor [16]. Cai et al. introduced single-walled carbon nanotubes into a polyvinyl alcohol (PVA) matrix to prepare highly elastic self-healing piezoresistive strain sensors, which could withstand 1000% elastic deformation and display fast electrical healing speed (<3.2 s) [17]. Jing and coworkers prepared a nanocomposited hydrogel comprised of polyacrylic acid and reduced graphene oxide, which presented high stretchability (600%), strong mechanical strength (400 kPa), and excellent self-healing property (75%) [18].

MXene (Ti_3_C_2_T_*x*_), a newly developed two-dimensional (2D) laminated transition metal carbide, has been widely proposed as an electrochemical energy storage material for its high electrical conductivity, excellent mechanical property, and large numbers of surface hydrophilic groups [19, 20]. It also shows promising prospect in piezoresistive sensing materials because the sliding and stacking of MXene nanosheets under tension or compression caused distinct variation in the number and length of conductive paths further leading to violent resistance variation [21]. For instance, Zhang and coworkers incorporated MXene into a commercial low-cost hydrogel, “crystal clay,” to monitor the direction of object movement due to its asymmetrical strain sensitivity [22]. Yue et al. used a simple dipping-coating process to obtain a 3D network MXene-sponge with wide pressure range (~18.56 kPa) and high sensitivity (442 kPa^−1^ within 5.37-18.56 kPa) [23]. However, it is still desirable to promote strategies to prepare MXene-based hydrogels with high stretchability and high sensitivity, as well as long durability.

Herein, a simple two-step synthesis strategy of a microwave-assisted aldol condensation reaction [24, 25] and freeze-thaw process [26] was proposed to synthesize a MXene-composited PVA/polyvinyl pyrrolidone (PVP) double-network hydrogel (MDN hydrogel). The resulting MDN hydrogel showed excellent mechanical properties of high stretchability, puncture resistance, and cyclic stability. The flexible sensors based on the MDN hydrogel successfully achieved high sensitivity, short response time, and high reproducibility to both tension and pressure. The hydrogel-based sensor also could accurately respond to different kinds of large human movements, as well as tiny physiological signal acquisition for health diagnosis. Moreover, it could be assembled into a sensor array to precisely detect the spatial pressure distribution, showing promising application for wearable electronics.

## 2. Results and Discussion

### 2.1. Design Principle and Material Synthesis

**Figure 1:**
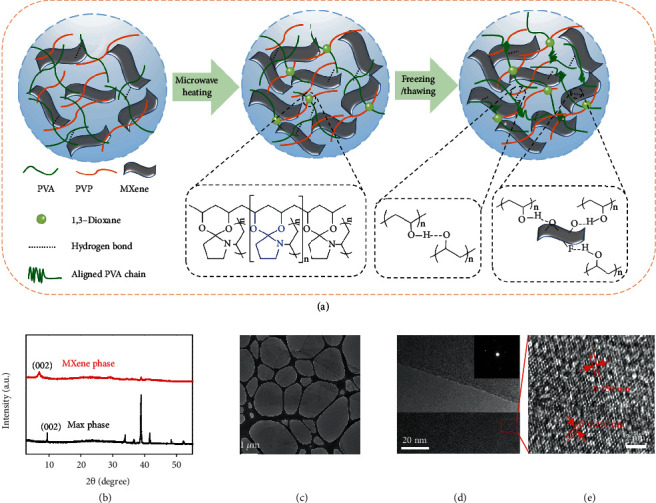
Design principle and material synthesis. (a) Schematic illustration of the synthesis route of the MXene-composited double-network hydrogel. (b) XRD patterns of MXene and Max phase. (c–e) TEM and HRTEM images of MXene and the corresponding diffraction pattern. Inset: SAED diffraction of MXene.

The MXene-composited hydrogel was prepared by incorporating MXene nanosheets into a PVA/PVP double-network hydrogel system. MXene nanosheets were obtained according to a modified hydrofluoric acid etching method [27].  Figure 1(b) shows the X-ray diffraction (XRD) patterns of the MAX phase and the resulting MXene nanosheets. The characteristic peak of MAX in the (002) plane at 9.5° shifts to a lower degree of 6.9° in MXene, indicating the successful formation of Ti_3_C_2_T_*x*_ nanosheets. The scanning electron microscopy (SEM) image suggests that the diameters of the MXene nanosheets are ranging from 0.5 to 6 *μ*m (Figure S1). As shown in Figure 1(c), the transmission electron microscopy (TEM) image indicates that the MXene sheets are large, ultrathin, and well dispersed. The selected area electron diffraction (SAED) pattern of the corresponding area, inserted in Figure 1(d), exhibits a standard hexagonal symmetric crystal, which is consistent with previously reported results. The high-resolution TEM (HRTEM) image also presents distinct lattice fringes. And the lattice spacing of 0.259 and 0.257 nm (Figure 1(e) and Figure S2) can be well indexed to the (0110) plane of the Ti_3_C_2_ phase.

Figure 1(a) schematically illustrates the synthesis process of the MXene-composited double-network hydrogel. Firstly, MXene nanosheets, PVA, and PVP were dissolved in deionized (DI) water under stirring to form a homogeneous solution. Afterwards, sulfuric acid solution was added and experienced an immediate reaction with the assistance of microwave. Under the catalysis of sulfuric acid, the alcoholic hydroxyl groups (-OH) of the PVA chain and the keto groups (C=O) of the PVP chain self-assembled into 1,3-dioxane polymer [25]. Fourier Transform Infrared (FTIR) spectra of PVA, PVP, and the double-network hydrogel (Figure S3) indicate that the characteristic peaks of PVA appear at 3273 cm^−1^ for -OH stretching vibration and 1316 cm^−1^ for C-O stretching vibration, respectively. And the characteristic peak of PVP appearing at 1661 cm^−1^ can be observed for the C=O stretching in the pyrrole ring. In comparison, the characteristic peak at 1657 cm^−1^ (C=O) decreases after the formation of the double-network hydrogel probably due to the acid-catalyzed ketalization reaction between -OH groups on the PVA chain and ketone groups on the PVP chain. Furthermore, a new peak appearing at 1079 cm^−1^ for the C-O-C stretching vibration of the formed 1,3-dioxane can be observed.

In the double-network hydrogel, PVP, a “hard segment” with larger side chain groups, is incorporated into the “soft segment” of PVA. The networked structure of the “soft” and “hard” segments makes the hydrogel exhibit excellent mechanical properties. The robust crosslinking of dioxane endows the hydrogel with high toughness and robustness. Hydrogen bonds, among PVA chains and between the polymer matrix and MXene nanosheets, are regarded as reversible interactions. The breaking and recombination of reversible bonds during stretching can favor the hydrogel excellent mechanical resilience. Finally, the composited solution was frozen at -20°C for 12 h and thawed at room temperature to obtain the double-network hydrogel. The freezing and thawing process could change the randomly curled PVA chain into locally ordered nanocrystalline domains, and the formed crystal segments could further enhance its mechanical strength [28].

### 2.2. Mechanical Properties of MDN Hydrogels

**Figure 2:**
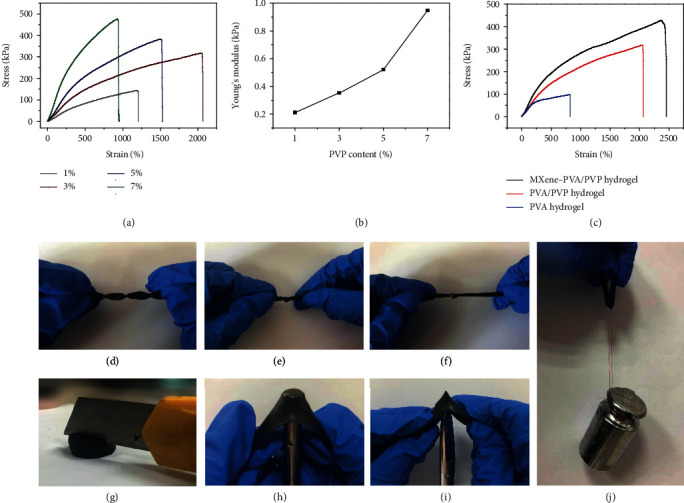
Mechanical properties of the MDN hydrogels. (a) Stress-strain curves and (b) Young's modulus of the double-network hydrogels with different contents of PVP (1, 3, 5, and 7 wt%). (c) Stress-strain curves of PVA hydrogel, double-network hydrogel, and MDN hydrogel. Diagrammatic sketch of the MDN hydrogel (d) twisting, (e) knotting, and (f) stretching after making a knot. The MDN hydrogel bears the sharp pressure of a knife (g), a metal cylinder (h), and scissors (i). (j) A sample loads 500 g weight.

To obtain the best mechanical properties of the double-network hydrogel, PVA/PVP hydrogels with different PVP contents were prepared and the stress-strain performances were measured. As shown in Figure 2(a), the tensile stress enhances with the increase in PVP content. Comparing Young's modulus of the double-network hydrogels within 0-250% strain, it demonstrates that the addition of PVP significantly improves the stiffness of the hydrogels (Figure 2(b)). In terms of the hydrogel stretchability, the 3 wt% PVP hydrogel exhibits the maximum tensile fracture length of 2055%, which is superior to reported double-network hydrogels in some literatures [11, 18, 29]. When the PVP content is further increased, the tensile fracture length begins to decrease. This phenomenon maybe comes from the synergistic effect of the polymer binary networks. And the addition of reinforcing components will improve the brittleness and toughness simultaneously. Therefore, there is an optimal proportion to balance the flexibility and robustness of the hydrogel. In this experiment, the double-network hydrogels with 3 wt% contents of PVP are designated for further research.

It is worth mentioning that experiments also reveal that the molecular weights of PVP also have great impacts on the mechanical performance of the hydrogel, and PVP with a molecular weight of 55000 exhibits the best performance, as illustrated in Figure S4. It suggests that PVP with smaller molecular weight will hinder the ketone reaction with the PVA chain, while PVP with excessive molecular weight will form a highly crosslinked entangling network with PVA chains and block the strong interaction within PVA chains. And the highly entangling network leads to significant decrease in the breaking strain and fracture strength of the double-network hydrogel. Figure 2(c) presents the mechanical properties of a single-network PVA hydrogel, double-network PVA/PVP hydrogel, and MDN hydrogel. The addition of MXene nanosheets (1 wt%) greatly increases the ductility and toughness of the hydrogel due to the substantial surface functional groups (-OH, -F, -O, etc.) of MXene nanosheets which can form hydrogen bonds with PVA chains to further enhance the mechanical strength (2400%) [19, 23]. Figure S5 shows five times stretching-relaxing cycle under 65% strain. The tensile strength decreases after the first stretching cycle due to inevitable viscosity of the polymer matrix and some permanently broken chemical bonds. And the subsequent stretching cycle curves coincide with each other, indicating that the breaking and recombination of reversible bonds are highly reproducible and stable.

Moreover, the obtained MDN hydrogel is highly flexible and robust to be curled, knotted, and stretched after knotting (Figures 2(d), 2(e), and 2(f)). Figure S6a shows that the MDN hydrogel can withstand large compression and recover to its initial state quickly after pressure is removed, presenting excellent mechanical resilience. Due to the large numbers of hydrophilic groups in the polymer matrix, the MDN hydrogel also exhibits a certain degree of adhesion, which can be closely attached to human skin (Figure S6b). More importantly, the MDN hydrogel shows strong toughness to accommodate local stress concentration. There is no crack or even a scratch on the surface of hydrogel after being pressed by a sharp blade of a knife (Figure 2(g) and Figure S6c). When the MDN hydrogel (thickness: 1.5 mm) is stretched under biaxial tension, neither a metal rod (Figure 2(h)) nor a scissor tip (Figure 2(i)) can easily pierce it, showing excellent puncture resistance. And the specific MDN hydrogel is tough enough. As shown in Figure 2(j), the strip with 3 mm in width and 2 mm in thickness can load 500 g weight without breaking.

### 2.3. MDN Hydrogel-Based Strain Sensor

**Figure 3:**
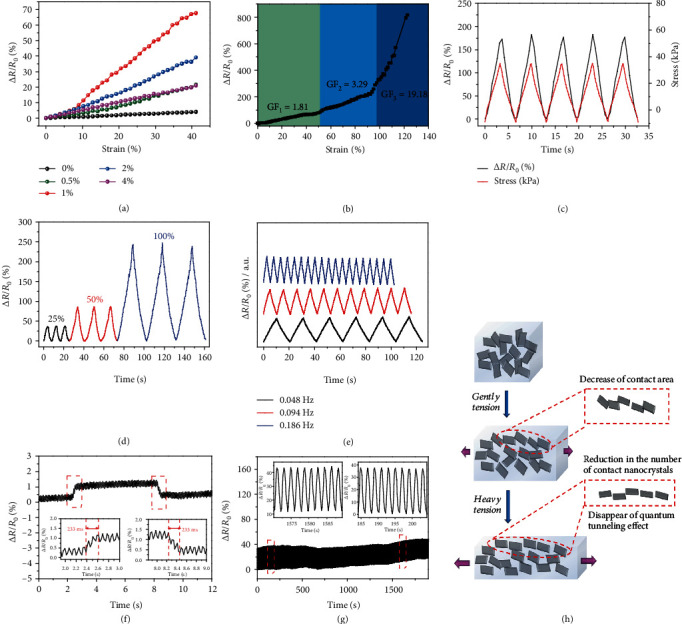
The electromechanical performances of the hydrogel-based strain sensor. (a) Δ*R*/*R*_0_ as a function of applied strains within 40% with different contents of MXene (0, 0.5, 1, 2, and 4 wt%). (b) Δ*R*/*R*_0_ as a function of applied strains up to 120% and the corresponding GF. (c) Electromechanical hysteresis of the hydrogel sensor. (d) Δ*R*/*R*_0_ under cyclic stretching-releasing at strains of 25, 50, and 100%, respectively. (e) Cyclic stretching-releasing with different stretch frequencies. (f) Response time and release time of the hydrogel sensor. (g) The durability test of the hydrogel sensor. (h) Schematic illustration of the mechanism of the electromechanical responses of the hydrogel sensor.

The sensitivity of a strain sensor is defined as a gauge factor (GF) and calculated by the formula: GF = (Δ*R*/*R*_0_)/*ε*, where Δ*R* = *R* − *R*_0_ and *R*_0_ and *R* are the raw resistance and the resistance under deformation, respectively. *ε* is the applied strain [15, 30, 31]. In the MDN hydrogel, the highly conductive MXene nanosheets form a 3D conductive network for electron conduction, presenting a predominant impact on the piezoresistive property. Sensing performance of the MDN hydrogel is evaluated by adjusting the content of MXene from 0 to 4% within 40% strain. As shown in Figure 3(a), GF increases with the rise in MXene content. When the MXene increases to 1%, GF reaches the highest value and then declines with the further addition of MXene. MXene nanosheets could build highly crosslinked conductive network, bringing about distinct resistance variation under tension. However, when excessive MXenes are incorporated, the accumulation of MXene nanosheets inevitably impedes their sliding under deformation, resulting in an obscure reduction in contact resistance and ultimately leading to a faint GF. Therefore, the optimal content of MXene in the double-network hydrogel is about 1%. As displayed in Figure S7, the MDN hydrogel is connected in series with a small LED and a battery. The brightness of the LED dims after stretching, which is consistent well with the resistance variation of the hydrogel.

As shown in Figure 3(b), the sensitivity of the strain sensor is 1.81 in the strain range of 0-50% and rises to 3.29 between 50% and 95%, which finally violently reaches 19.18 when higher strain is applied (95-120%). In comparison, the double-network PVA/PVP hydrogel (without MXene) sensor shows negligible electrical response to strains in the range of 0-40% (Figure 3(a)), exhibiting a quite dreadful GF value. It is considered that the MXene nanosheets play an extremely important role to improve the sensitivity of the double-network hydrogel. The possible mechanism of the three distinct GFs under different stain ranges is depicted in Figure 3(h). The resistance of an MXene-based 3D conductive network is composed of island resistance, gap resistance, and contact resistance [32, 33]. Island resistance refers to the intrinsic resistance of MXene and remains constant under deformation. Under a small strain, the stacking of the MXene nanosheets maintains a continuous conductive network throughout the hydrogel. And the Poisson effect of the polymer matrix increases the length for electron conduction, leading to a linear response to the applied strain within 0-40% with a low GF. When the hydrogel is further stretched, the contact area of the MXene nanosheets decreases gradually, making a marked increase in contact resistance and a rapid increase in GF. When larger tensile strain is further applied, cracks between MXene nanosheet domains propagate and the direct contact among nanosheets is lost; consequently, the tunneling effect between the edges of the cracks dominates the electron conduction in hydrogel [34]. With a significant decline in the number of conducting paths, the tunneling resistance sharply rises with the ascending tension and achieves a highly enhanced GF.

Figure 3(c) compares the output relative resistance variation with the input signal of stress under 60% strain. Two waveforms are almost synchronized with each other, indicating that the electromechanical hysteresis is negligible. Figure 3(d) shows the relative resistance change of an MDN hydrogel strain sensor under strains ranging from 25% to 100%. The relative resistance change (Δ*R*/*R*_0_) increases with the applied strain in three consecutive cycles, which suggests that the strain sensor can monitor different levels of tensile strains with excellent reliability. Figure 3(e) shows the frequency response and output signals of the MDN hydrogel strain sensor under a strain of 16%. It can be observed that the resistance variations of the sensor are reproducible and durable in the frequency range of 0.048 to 0.186 Hz. This phenomenon can be attributed to the highly crosslinked dual network and the strong interaction between MXene nanosheets and the polymer matrix. Besides, the amplitude of the variations in resistance is analogous at different frequencies, suggesting a typical frequency-dependent behavior. The response time of the hydrogel sensor under stretching and releasing is shown in Figure 3(f). It presents a rapid response to external stimuli (3%) with a short response time of 233 ms, ensuring a fast response to instant human motions. Figure 3(g) shows relative resistance changes of the MDN hydrogel sensor during a sequent 1000 stretching and releasing cycles during 0~25% strain. Actually, the resistance variation is constant during the entire durability tests as verified by the inset enlarged curves in Figure 3(g); the water loss during the testing process was calculated to be 2.1%, demonstrating excellent reproducible and stable durability of the strain sensor. Meanwhile, it appears that the baseline gradually increases with cycles, which may be attributed to the inevitable viscosity of polymer matrix and evaporation of water in the hydrogel.

### 2.4. MDN Hydrogel-Based Pressure Sensor

**Figure 4:**
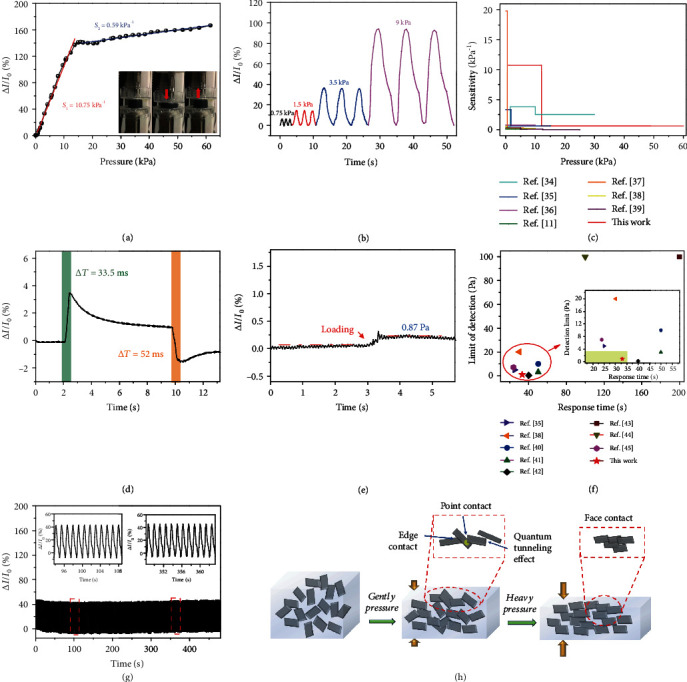
The electromechanical performances of the hydrogel-based pressure sensor. (a) Relative current variation versus pressure for the hydrogel sensor. (b) Relative current variation as a function of time under different pressures (0.75, 1.5, 3.5, and 9 kPa). (c) Comparison of sensitivity and pressure range with literatures. (d) Response time and release time of the hydrogel sensor. (e) Detection limitation of the hydrogel sensor. (f) Comparison of detection limitation and response time with literatures. (g) The durability test of the hydrogel sensor. (h) Schematic illustration of the mechanism for the electromechanical responses of the hydrogel sensor.

As shown in Figure S6a, the MDN hydrogel also exhibits excellent mechanical resilience to pressure, showing potential application in the field of pressure sensors. Herein, a specific cylindrical MDN hydrogel with a diameter of 14 mm and a height of 8 mm was assembled into a pressure sensor. The compression stress-strain curves of the pressure sensor are shown in Figure S8. The hydrogel can be compressed to the extreme (47% strain) with a maximum pressure of 65 kPa. Sensitivity, which indicates the electrical response upon pressure, is defined as *S* = *δ*(Δ*I*/*I*_0_)/*δP*, where *I*_0_ is the current without pressure, Δ*I* is the current variation with certain pressure, and *P* is the pressure loading.  Figure 4(a) displays the current variation curves under different loading pressures. It can be distinguished that the Δ*I*/*I*_0_ undergoes a sharp increase below 13.8 kPa and a gentle enhancement above 13.8 kPa, exhibiting two distinct regions within a wide sensing range of 0-61.5 kPa. By calculation, the sensitivity of the MDN hydrogel pressure sensor is 10.75 kPa^−1^ below 13.8 kPa and 0.59 kPa^−1^ in the range of 13.8-61.5 kPa.  Figure 4(h) schematically displays the possible movement of the MXene nanosheets under compression. It is speculated that when a gentle pressure under 13.8 kPa is applied, the distance between adjacent MXene nanosheets decreases rapidly, and the loosely distributed MXene nanosheets form a direct point contact or edge contact, causing a significant decline in resistance. In addition, when the spacing of the adjacent MXene nanosheets is smaller than the tunneling distance, the quantum tunneling effect takes effect and leads to a further decrease in resistance. When imposing to a larger pressure above 13.8 kPa, most of the MXene nanosheets, already being in a direct contact, are more orderly arranged with a gentle increase in the contact area between nanosheets, resulting in a mild decrease in resistance. In Figure 4(b), the relative current changes of the MDN hydrogel pressure sensor under different pressures of 0.75, 1.5, 3.5, and 9 kPa are measured to be 6.1, 14.3, 36.3, and 94.3, respectively, which is highly consistent with the results in Figure 4(a). The pressure sensor combines the advantages of high sensitivity at low pressures and a wide response range, which is superior to some reported hydrogel-based pressure sensors (Figure 4(c)) [11, 35–40].

The highly crosslinked MXene conductive dual network also endows the pressure sensor with extremely short response time (33.5 ms) and recovery time (52 ms) for a stress stimulus of 800 Pa (Figure 4(d)), which exceeded many reported hydrogel-based pressure sensors [41–43]. As shown in Figure 4(e), when a piece of paper (0.1462 g) with an area of 14 × 12 mm^2^ is loaded onto the surface of the pressure sensor, the relative current variation increases by one step and stabilizes thereafter, indicating a susceptive response to subtle pressure. The limit of detection of the pressure sensor is measured to be 0.87 Pa. Compared with literatures [36, 39, 41–46], the MDN hydrogel pressure sensor demonstrates extraordinary sensing performances in terms of response time and detection limitation (Figure 4(f)).  Figure 4(g) shows the durability of the MDN hydrogel pressure sensor at a cyclic pressure loading of 0-4 kPa. The current variations are almost the same for 380 loading/unloading cycles, suggesting that the pressure sensor has predominant durability and stability, and it is more suitable for practical application.

### 2.5. Application in Human Motion Detection

**Figure 5:**
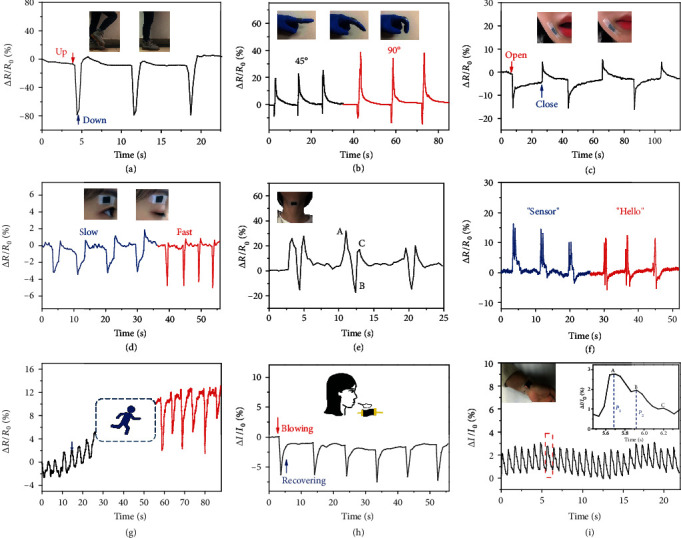
Human motion signal detection. Relative resistance changes of the hydrogel sensors during (a) bending of a knee joint, (b) bending of a finger at a certain angle (45° and 90°), (c) opening and closing the mouth, (d) blinking an eye, (e) swallowing movement, (f) speaking in response to similar sounds of “sensor” and “hello,” (g) different breath detections before and after running, (h) blowing on the surface of the sensor (26°C, RH: 60%), and (i) beating of a human pulse.

The MDN hydrogel sensor, with excellent stretchability, sensitivity, and durability to both tension and compression, provides an alternative approach to flexible electronic skin. To explore its application in physiological activities, the sensor is applied to identify various large-scale and subtle human motions. As shown in Figure 5(a), the MDN hydrogel sensor is attached on the knee to monitor the leg movement, and the resistance shows a sharp decreased response to the leg lifting. Figure S9a shows the relative change in resistance when bending the elbow horizontally, and Figure S9b presents the electrical response of ankle movements. The relative changes in resistances show clear peaks, sharp changes, and stable responses for monitoring large-scale human motions, which confirms that the sensor has a fast response and high robustness. In Figure 5(b), a small piece of a sensor is attached to the index finger to monitor finger flexion. When the finger is bent at a certain angle, the variation of the relative resistance changes uniformly for three consecutive cycles, and the peaks of the relative resistance are more distinct when the angle increases from 45° to 90°, which proves that it can accurately track the angle of the finger bending. Figure 5(c) shows a distinct rise and decline in relative resistance when the mouth is opened and closed. When the sensor is attached to the eyelid (Figure 5(d)), typical vibration on the relative resistance at different frequencies can be clearly distinguished to indicate the slow and fast blinking, showing a wide prospect for facial recognition.

As shown in Figure 5(e), the hydrogel sensor is pasted onto the neck to monitor the throat movements during swallowing. The measured relative resistance variation includes three characteristic peaks, which is consistent with the theoretical change in resistance of the swallowing action [47, 48]. The first peak corresponds to the movement of the back of the tongue against the hard palate, and the water is pushed towards the pharynx behind the soft palate. Then, a downward characteristic peak appears in the curve because the passage of the trachea is closed, and water is squeezed from the pharynx into the esophagus. Finally, when the water enters the esophagus, it causes the esophagus to move and pushes water into the stomach through the cardia, resulting in another upward peak. The sensors can accurately distinguish vibration signals between two vocal cords. When speaking English words, such as “sensor” and “hello,” distinguishable and reproducible signal patterns are yielded, exhibiting promising application for phonetic recognition (Figure 5(f)).

The sensor also can be utilized to detect subtle psychological information of the human body. Figure 5(g) depicts the relative resistance change with abdominal muscle movement caused by breathing when the sensor is attached to the abdomen of a human body. The change value of relative resistance during shallow breathing was 3.92% and the frequency was 0.26 Hz, while the change value of relative resistance during deep breathing was 10.27% and the frequency was 0.20 Hz. Compared with the shallow breath before running, the curve of relative resistance after running presents a stronger signal strength and a lower frequency, representing a deeper breath. Furthermore, the sensor can be used to detect changes in airflow or human breathing (Figure 5(h)).

When the sensor is attached to the beating position of the artery on the wrist, the regular beat of the pulse can be clearly identified. As can be seen from Figure 5(i), each period of relative current change is 789 ms, and a simple conversion can obtain 76 beats per minute, which is a normal person's heartbeat frequency. The inset picture shows that the peak of a single arterial pressure wave displays three subtle component peaks: radial artery pulse waveform (denoted as “a” and “c”) and systolic augmentation shoulder (denoted as “b”). The value of P2/P1 (peak height ratio) represents radial dilation index (AI), which can be used to determine the degree of arteriosclerosis [49, 50]. The AI value calculated from the graph is 0.706, which is an expected value for a healthy female. So, the MDN hydrogel sensor is highly expected for portable and facile vascular health monitoring.

### 2.6. Application of Pressure Sensors

**Figure 6:**
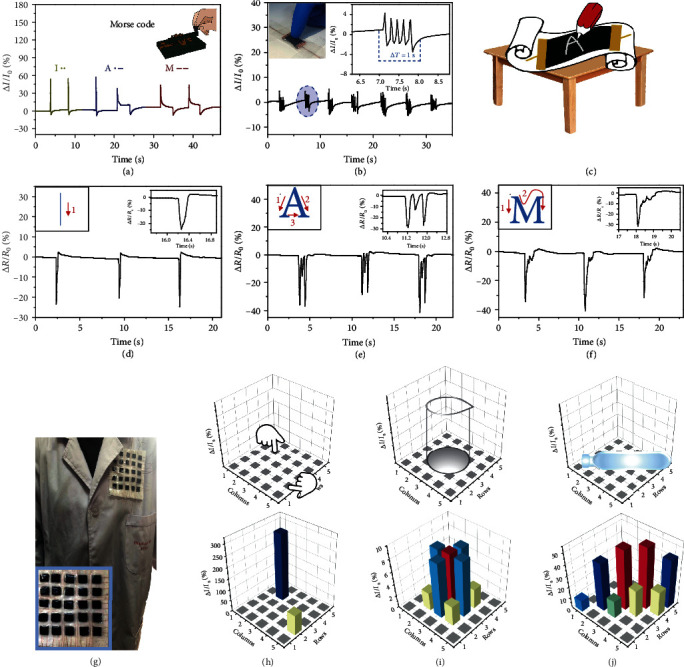
Application of pressure sensors. (a) Simulation of Morse code for “IAM” produced by tapping the hydrogel sensor. (b) Sensing performance of the hydrogel sensor under imitated knocking of early-stage Parkinson's disease. Inset: amplified current variation. (c) Schematic for writing sensing. (d–f) Writing sensing of different English letters (“I,” “A,” and “M”). (g) Photographs of the device for position detection. The relative current change distribution of (h) finger press stresses, (i) a beaker, and (j) a centrifuge tube filled with water.

Due to the high sensitivity with wide sensing range, short response time, and low detection limitation, the MDN hydrogel pressure sensor has been applied to some tiny stimulation and characteristic pressure detections. Morse code is a classic signal code to express different English letters through arrangements of dots and horizontal lines, in which a quick tap represents a point and a sustained press represents a horizontal line. As shown in Figure 6(a), by tapping or pressing the sensor, the output relative current change curves can simulate the Morse password of three letters “I,” “A,” and “M” precisely and promptly. Because of the fast response and recovery characteristics of the MDN hydrogel sensor, it also can be applied to the field of static vibration. By high-frequency tapping a pressure sensor, distinct and durable current fluctuation is achieved, as shown in Figure 6(b). The enlarged image shows a tapping frequency of 5 Hz, which means a potential application of the sensor for early Parkinson's disease prediction (manifesting as rhythmic tremor 4-6 times per second). Writing is a complex stress stimulus, including movement directions, writing strength, and speed. During the measurements, different letters are written on the pressure sensor. In view of different letters having different numbers and directions of strokes, the response signals show different peak shapes. As shown in Figure 6(d), the sensing curve of the letter “I” exhibits just one peak in terms of only one stroke, while three distinct peaks can be distinguished for the letter “A,” which has three strokes (Figure 6(e)). The strokes of the letter “M” are more complex and are shown in detail in the sensing curve (Figure 6(f)). The precise sensing of letter strokes reflects the huge potential of the hydrogel sensor for handwriting verification and anticounterfeiting applications.

To demonstrate that the MDN hydrogel can be used for large-scale and unevenly distributed stress monitoring, a 5 × 5 dot matrix device (composed of 25 square hydrogels with a side length of 1 cm) is assembled. Due to the hydrophilicity of the hydrogels, the dot matrix device can be assembled on a flexible substrate, for example, a piece of gauze. Therefore, it can be directly sewn on the clothing (Figure 6(g)). The flexible and dense dot matrix enables the device to detect stress stimuli in two different ways, “point” and “flat.” As shown in Figure 6(h), a “point” pressure is applied by a finger touch and the obtained 3D image precisely responds to the magnitude and position of the pressure, making a base for matter perception with complex shapes. When an empty glass beaker is placed on the device, the weight of the beaker causes a local circular “flat” pressure. A 3D image is output through gathering relative current changes in the matrix, which graphically represents the spatial pressure distribution (Figure 6(i)). In the same way, a centrifuge tube filled with water is placed on the surface of the device and the output signal perfectly shows the pressure distribution (Figure 6(j)). It can be concluded that the aligned matrix pressure sensor demonstrates excellent sensitivity in spatial unevenly distributed pressure differentiation, which indicates promising potential for application in wearable electronics.

## 3. Conclusion

In summary, MXene-composited double-network hydrogels with excellent mechanical performances and conductivity have been successfully constructed by integrating MXene into PVA/PVP hydrogels. The strong interactions between PVA and PVP chains and hydrogen bonding endow the hydrogel with high stretchability (2400%), high toughness, and excellent stress tolerance. Meanwhile, the strain sensor based on the MXene-composited double-network hydrogel exhibits high sensitivity (GF = 19.18), timely response, and excellent durability. The hydrogel-based pressure sensors also present outstanding sensing performances including high sensitivity (10.75 kPa^−1^) within wide pressure sensing range, extremely fast response time (33.5 ms), and low detection limitation (0.87 Pa). The fabricated hydrogel sensors can realize real-time monitoring of multiple physiological stimuli on human activity, such as knee flexion, finger bending, speech and pulse vibration, and health diagnosis. It can further be assembled into a complicated matrix for aligned pressure sensors, showing a great potential in human-machine interactions or other field applications.

## 4. Material and Methods

### 4.1. Materials

Poly(vinyl alcohol) (PVA, MW ~61000) and poly(vinylpyrrolidone) (PVP, MW ~55000) were purchased from Sigma-Aldrich Co. Other PVP (MW ~8000, 24000, 130000) was purchased from Aladdin Co. Sulfuric acid (98%) and hydrochloric acid were purchased from Shanghai Lingfeng Chemical Reagent Co., Ltd. Ti_3_AlC_2_ (powder, 200 meshes) was purchased from Beijing Forsman Scientific (China). Tetramethylammonium hydroxide (TMAOH, 25 wt% in water) was purchased from Admas. All the chemicals were used directly without further purification.

### 4.2. Synthesis of MXene Nanosheets

MXene was prepared via an etching method based on literature [27]. Briefly, 1.00 g Ti_3_AlC_2_ powder and 1.00 g LiF were dissolved in 10 mL HCl solution (9 M). After removing oxygen by injecting nitrogen, the mixture was sealed in an oven at 200°C for 24 h. Then, the obtained suspension was collected and washed. After dispersing the sediment in 10 mL TMAOH solution, Ti_3_C_2_ was collected by further centrifugation and washing. Eventually, Ti_3_C_2_ nanosheet powders were obtained by drying the supernatant with a freeze-drying method.

### 4.3. Preparation of MXene Double-Network Hydrogels

Firstly, PVA solution was prepared by dissolving 3.6 g PVA in 11.4 mL deionized water at 100°C for 1 h. Then, the PVA solution was mixed with 3 mL PVP solution (20 wt%), followed by stirring at room temperature. The uniform solution was poured into a glass beaker (100 mL), and 1 mL sulfuric acid (10 wt%) was added. The uniform solution was exposed to microwave (energy at 600 W) for 2 min using UWave-1000 (Shanghai Sineo). Afterwards, the MXene solution (10 mg/mL) was added into the mixture. Finally, the MDH hydrogel was frozen in a refrigerator at -20°C for 12 h and thawed at room temperature for 6 h with consecutive three cycles. The double-network PVA/PVP hydrogels were also prepared without MXene solution incorporation.

### 4.4. Mechanical Tests

The mechanical performances of the hydrogels were measured by a mechanical testing apparatus (ESM302, Mark-10). For the tensile tests, the hydrogels were cut into regular strips of 30 × 3 × 2 mm and clamped to the two ends of the stretching machine (stretching speed 60 mm/min). All the tensile tests were run at room temperature, and the specimens were prepared and tested immediately to prevent moisture loss. Tensile strength and elongation at break were determined by the breaking point. The elastic modulus was determined by the slope of the stress-strain curve in the strain range of 0-250%. For the pressure sensor, a specific cylindrical hydrogel with a diameter of 14 mm and a height of 8 mm was also assembled and tested.

### 4.5. Water Loss Measurements

The water loss measurements were performed to characterize the hydrogel dehydration performance at an ambient environment. Circular samples (diameter *Φ* = 20 mm and height *h* = 15 mm) were placed in the air for approximately 48 h. The ambient humidity is 52%, and the ambient temperature is 32°C. The water loss rate (*Q*) can be calculated by the following equation:

(1) $$\begin{array}{c} Q=\frac{W_{0}-W_{\text{t}}}{W_{0}}\times 100\%, \end{array}$$

where *W*_0_ is the weight of the hydrogel before the dehydration experiment and *W*_t_ is the weight after the dehydration experiment.

### 4.6. Electrical Tests

The resistance variations of the hydrogels were detected by a semiconductor characterization system (Keithley 4200-SCS). The hydrogels were secured to the mechanical testing apparatus when testing resistance changes in tension and compression. Each end of the hydrogel was inserted with copper wires to connect to Keithley 4200-SCS and further encapsulated to minimized environmental disturbance. For human body movement testing and other measurements, the hydrogels were completely encapsulated and taped to the appropriate area.

## Data Availability

All data needed to evaluate the conclusions in the paper are presented in the paper and/or the Supplementary Materials. Additional data related to this paper may be requested from the authors.

## Supplementary Materials

Supplementary Materials Supplementary 1. Figure S1: SEM images of MXene flakes. Figure S2: measured lattice fringes of MXene in HRTEM image. Figure S3: FTIR spectra of PVA, PVP, and the double-network hydrogel. Figure S4: mechanical properties of the double-network hydrogels with different molecular weights of PVP. Figure S5: stress-strain curves of five stretching-relaxing cycles for the hydrogel sensor. Figure S6: (a) withstand huge pressure and recover, (b) attached to the skin surface, and (c) undamaged surface after a knife cut. Figure S7: brightness of the small LED changes after stretching. Figure S8: compression stress-strain curves of the MDH hydrogel pressure sensor. Figure S9: relative change in resistance when bending the (a) elbow and (b) ankle.
