# Supplementary material for: Highly Stretchable, Elastic, and Sensitive MXene-Based Hydrogel for Flexible Strain and Pressure Sensors
Source: Research (Wash D C). 2020 Jul 14;2020:2038560. doi: 10.34133/2020/2038560 (PMC7376495; doi:10.34133/2020/2038560)
Supplement: Supplementary Materials — Supplementary 1. Figure S1: SEM images of MXene flakes. Figure S2: measured lattice fringes of MXene in HRTEM image. Figure S3: FTIR spectra of PVA, PVP, and the double-network hydrogel. Figure S4: mechanical properties of the double-network hydrogels with different molecular weights of PVP. Figure S5: stress-strain curves of five stretching-relaxing cycles for the hydrogel sensor. Figure S6: (a) withstand huge pressure and recover, (b) attached to the skin surface, and (c) undamaged surface after a knife cut. Figure S7: brightness of the small LED changes after stretching. Figure S8: compression stress-strain curves of the MDH hydrogel pressure sensor. Figure S9: relative change in resistance when bending the (a) elbow and (b) ankle. [file 2038560.f1.pptx]

## Slide 1
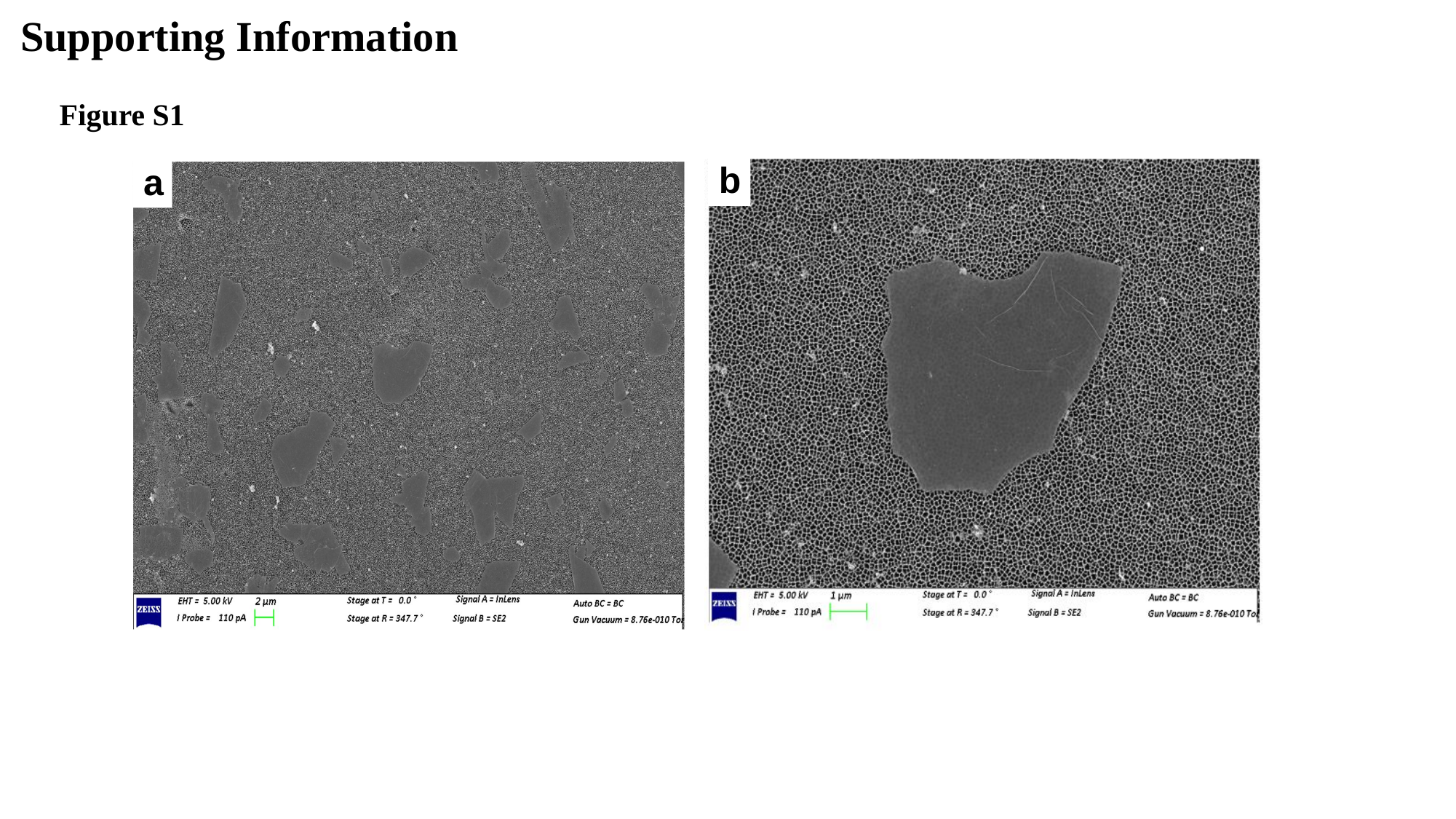

Supporting Information
Figure S1
a
b

## Slide 2
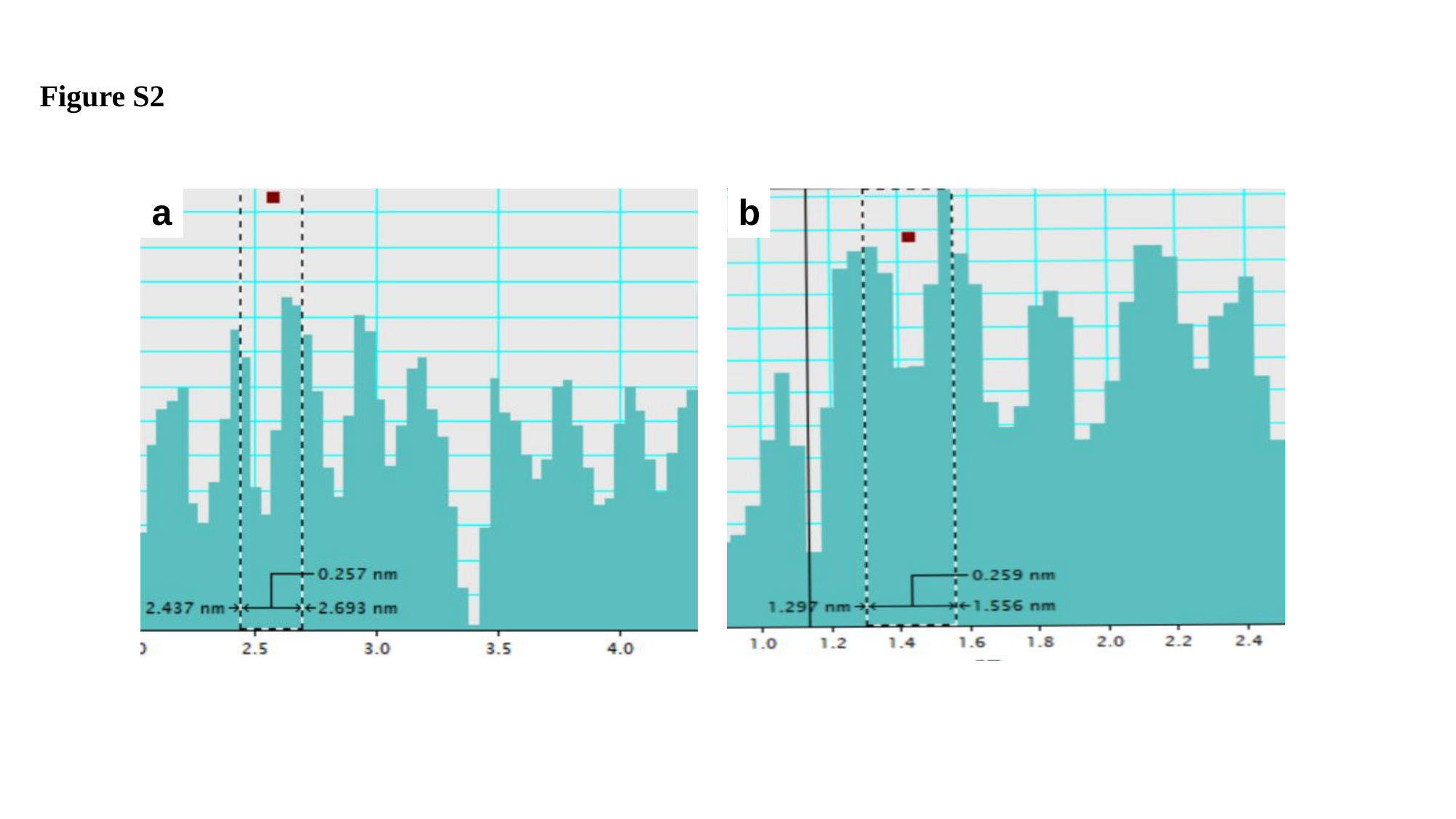

Figure S2
a
b

## Slide 3
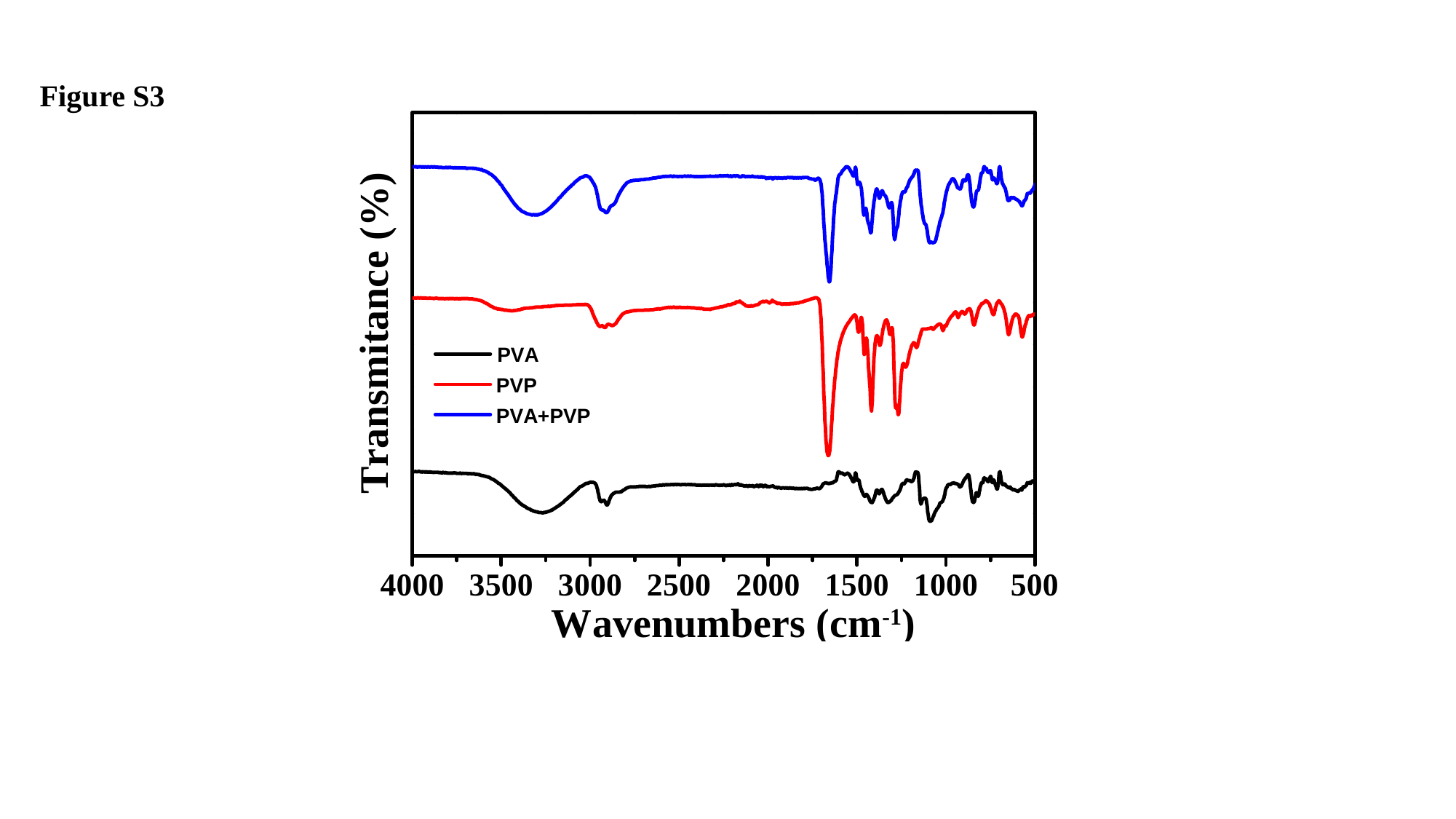

Figure S3

## Slide 4
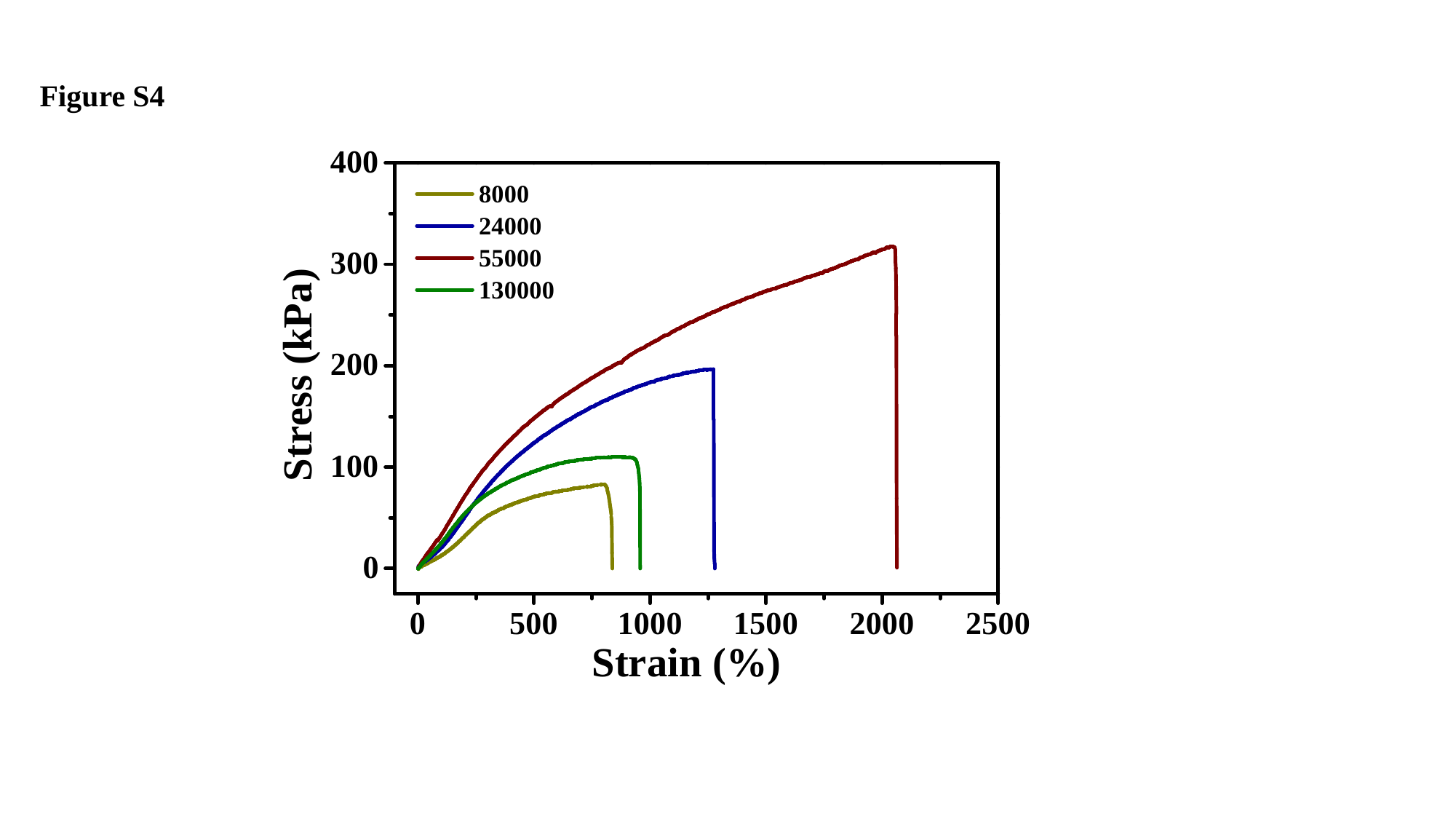

Figure S4

## Slide 5
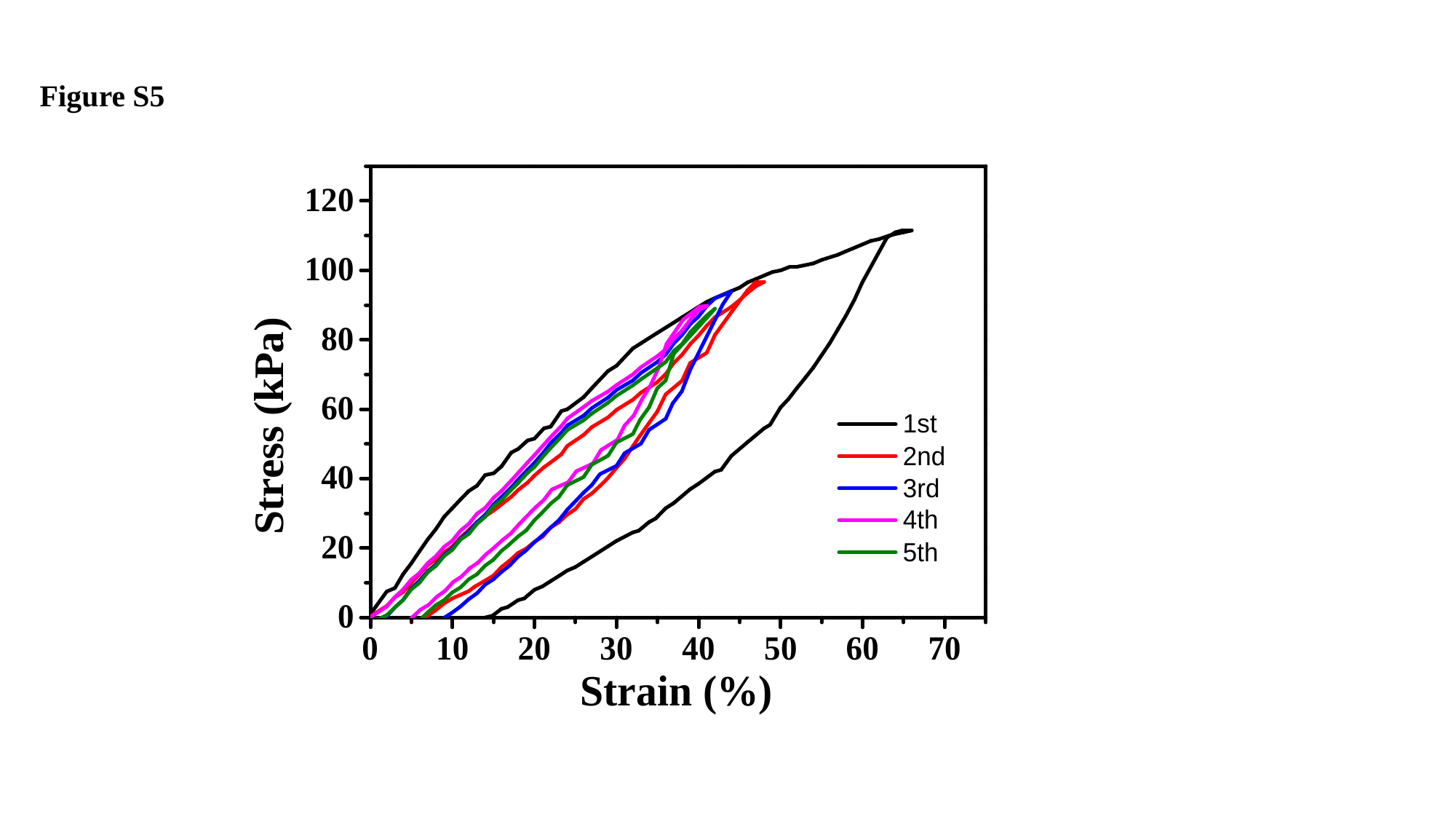

Figure S5

## Slide 6
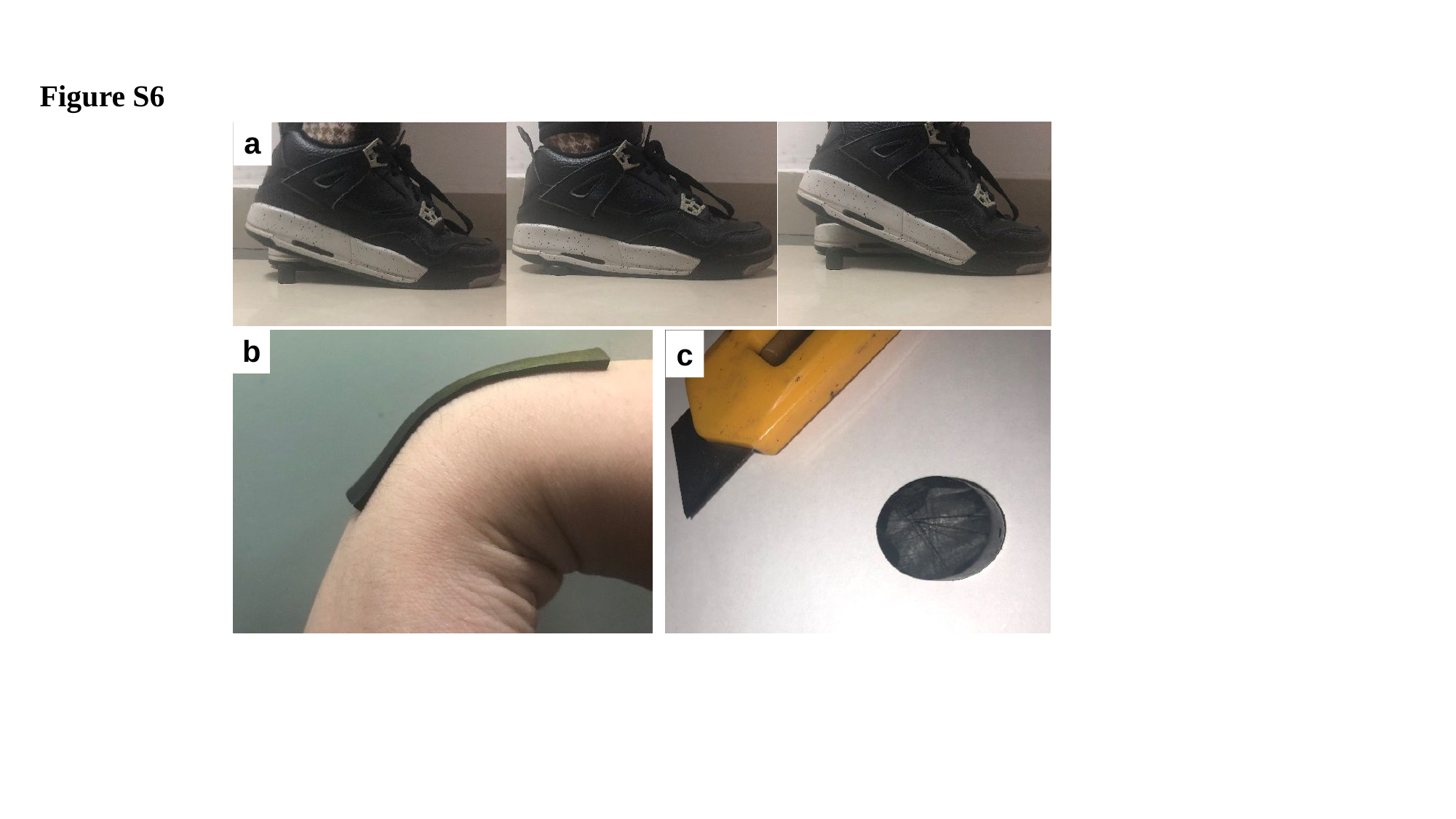

Figure S6
a
b
c

## Slide 7
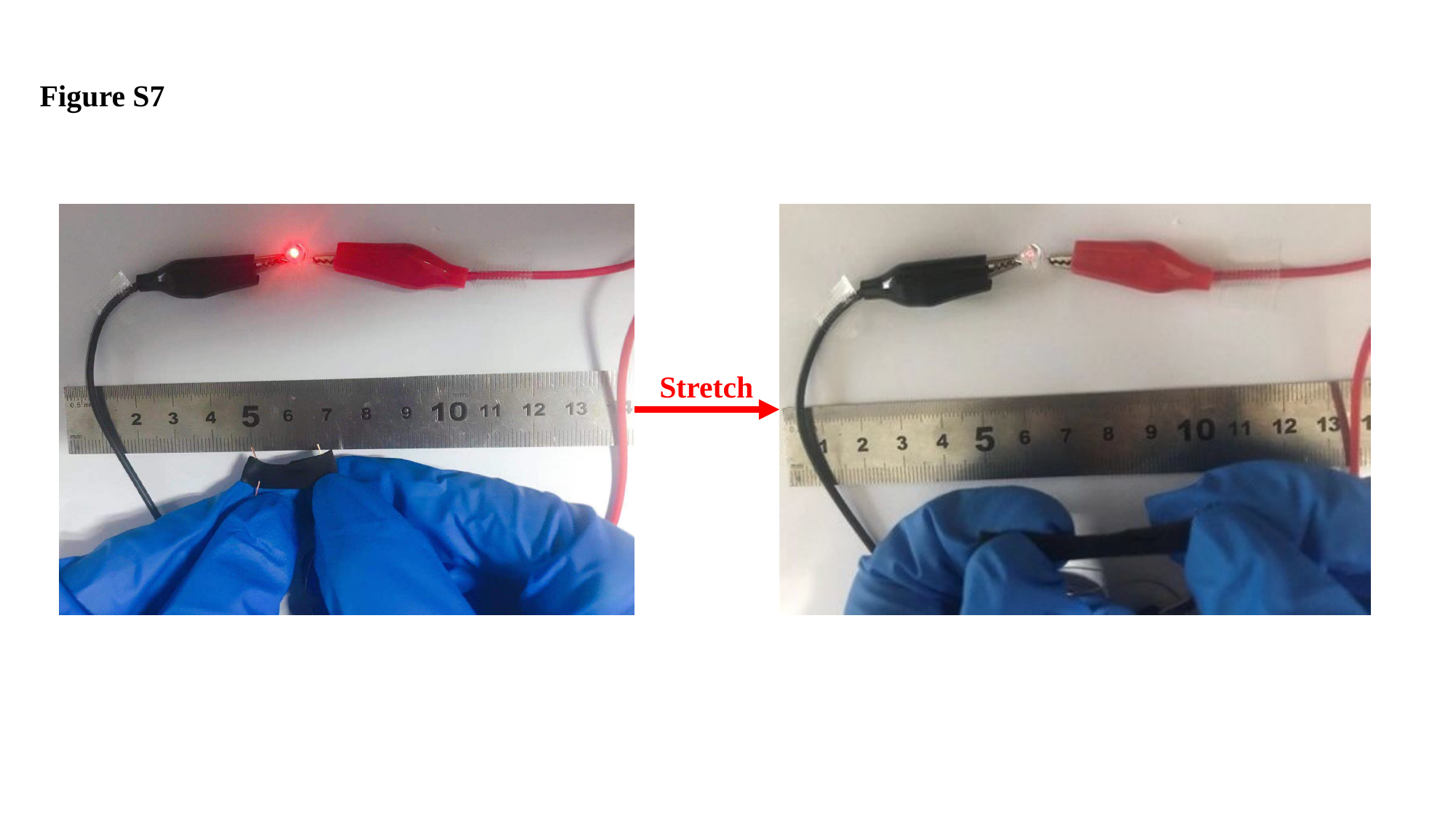

Figure S7
Stretch

## Slide 8
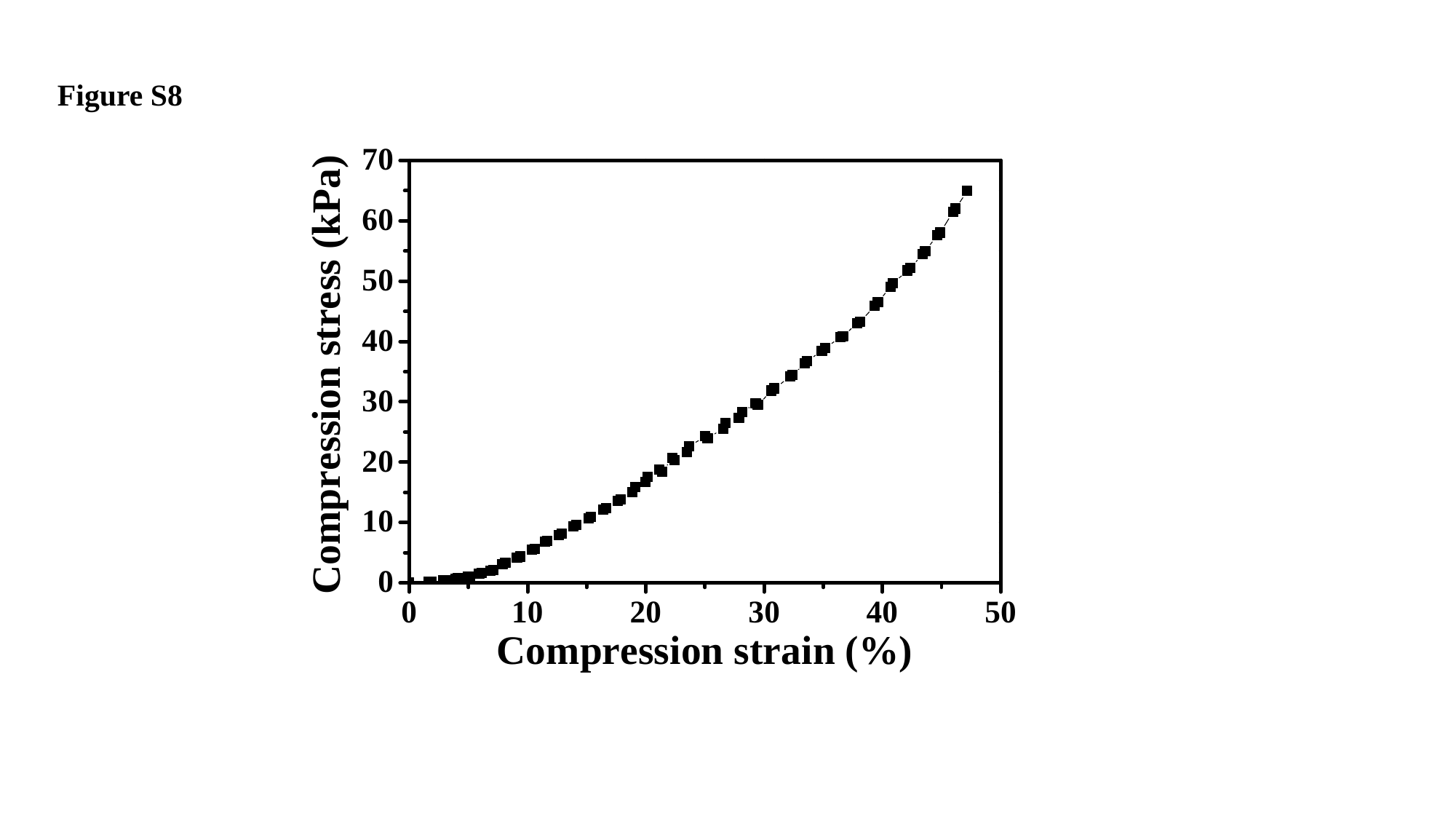

Figure S8

## Slide 9
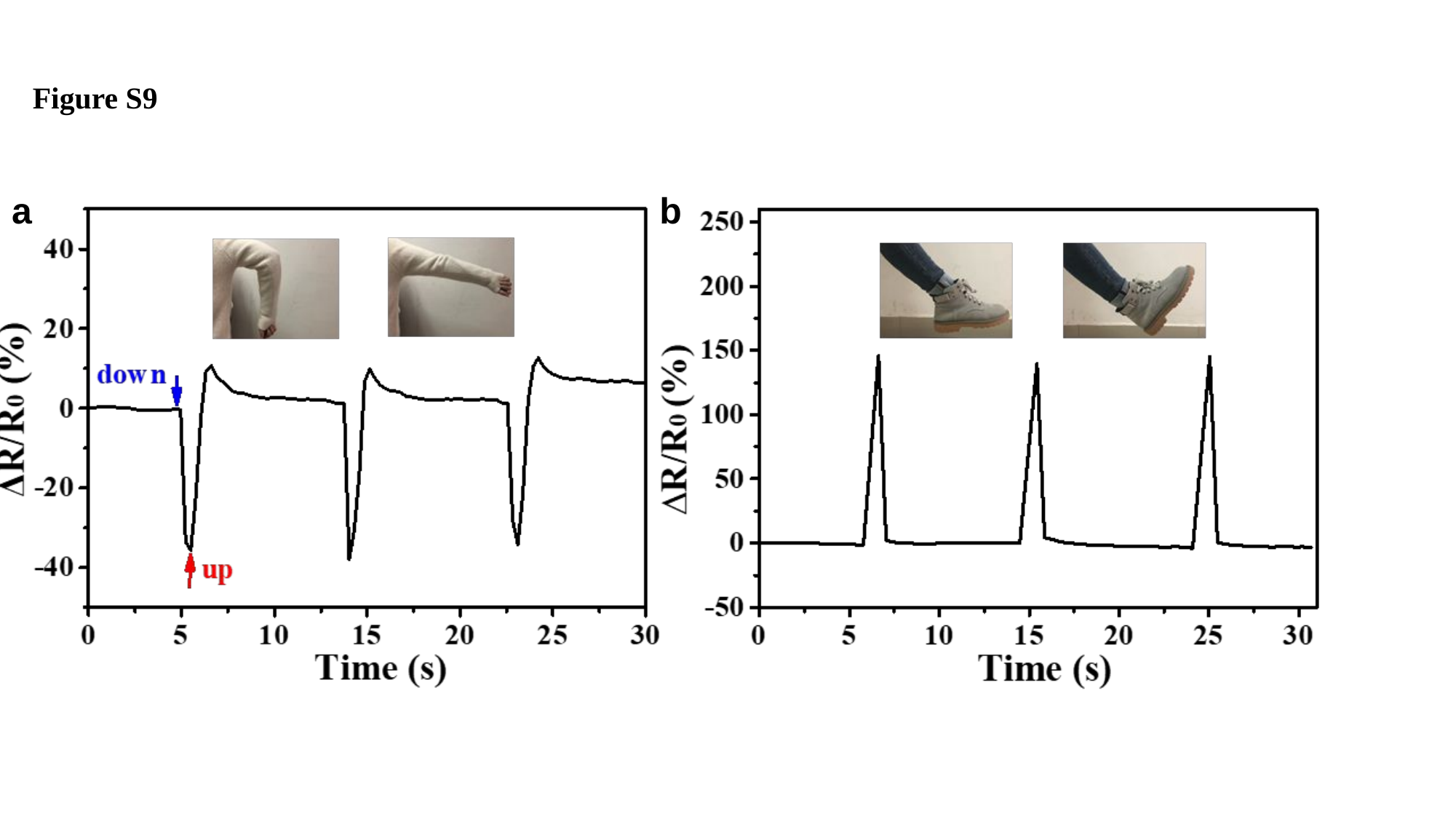

Figure S9
a
b
